# Crystal structure of a low-spin poly[di-μ_3_-cyanido-di-μ_2_-cyanido-bis­(μ_2_-2-ethyl­pyrazine)­dicopper(I)iron(II)]

**DOI:** 10.1107/S2056989019009496

**Published:** 2019-07-19

**Authors:** Sofiia V. Partsevska, Dina D. Naumova, Igor P. Matushko, Olesia I. Kucheriv, Il’ya A. Gural’skiy

**Affiliations:** a Department of Chemistry, Taras Shevchenko National University of Kyiv, Volodymyrska St 64, Kyiv 01601, Ukraine; b UkrOrgSyntez Ltd, Chervonotkatska St 67, Kyiv 02094, Ukraine

**Keywords:** crystal structure, ethyl­pyrazine, di­cyano­cuprate, iron(II), copper(I), bimetallic, metal–organic framework

## Abstract

In the title metal–organic framework, [Fe(C_6_H_8_N_2_)_2_{Cu(CN)_2_}_2_]_*n*_, the low-spin Fe^II^ ion lies at an inversion centre and displays an elongated octa­hedral [FeN_6_] coordination environment. The axial positions are occupied by two symmetry-related bridging 2-ethyl­pyrazine ligands, while the equatorial positions are occupied by four N atoms of two pairs of symmetry-related cyanide groups. The Cu^I^ centre is coordinated by three cyanide carbon atoms and one N atom of a bridging 2-ethyl­pyrazine mol­ecule, which form a tetra­hedral coordination environment. Two neighbouring Cu atoms have a short Cu⋯Cu contact [2.4662 (7) Å] and their coordination tetra­hedra are connected through a common edge between two C atoms of cyanide groups. Each Cu_2_(CN)_2_ unit, formed by two neighbouring Cu atoms bridged by two carbons from a pair of μ-CN groups, is connected to six Fe^II^ centres *via* two bridging 2-ethyl­pyrazine mol­ecules and four cyanide groups, resulting in the formation of a polymeric three-dimensional metal–organic coordination framework.

## Abstract 2

In the title compound, {Fe(Etpz)_2_[Cu(CN)_2_]_2_}_*n*_, where Etpz is 2-ethyl­pyrazine, the low-spin Fe^II^ atom lies on an inversion centre and has an elongated octa­hedral [FeN_6_] coordination environment, where the axial positions are occupied by two 2-ethyl­pyrazine ligands and the equatorial positions are occupied by two pairs of symmetry-related cyanido groups. Each Cu_2_(CN)_2_ unit is connected to six Fe^II^ centres *via* two bridging 2-ethyl­pyrazine mol­ecules and four cyanido groups, resulting in the formation of a polymeric three-dimensional bimetallic metal–organic framework, additionally stabilized by Cu⋯Cu contacts.

## Chemical context   

The phenomenon of spin crossover (SCO) occurs in some metal complexes where the spin state of a compound changes as a result of the influence of external stimuli (temperature, pressure, light irradiation, magnetic field *etc.*) (Gütlich & Goodwin, 2004 ▸). Analogues of Hofmann clathrates (Hofmann & Höchtlen, 1903 ▸) are the most diverse SCO compounds with switchable properties because of their specific structural features. They are bimetallic two- or three-dimensional coord­ination frameworks formed by Fe^II^ ions coordinated by cyano­metallic anions [*M*(CN)_*x*_]^*y*−^ and N-donor heterocyclic ligands (Ohkoshi *et al.*, 2014 ▸; Muñoz & Real, 2011 ▸). Such frameworks have been prepared in forms of single crystals, thin films (Bell *et al.*, 1994 ▸) and nanoparticles (Volatron *et al.*, 2008 ▸), thus presenting a group of materials characterized by the presence of sharp and hysteretic SCO. A large variety of Hofmann-like polymeric SCO complexes originates from a set of available cyano­metallates (formed by Ni, Pt, Pd, Ag, Au, Cu and Nb) and organic ligands, which potentially could promote the spin state change of Fe atoms (Muñoz & Real, 2011 ▸). Pyridine (Kitazawa *et al.*, 1996 ▸), amino­pyridine (Liu *et al.*, 2015 ▸), pyrazine (Niel *et al.*, 2001 ▸), azo­pyridine (Agustí *et al.*, 2008 ▸), pyrimidine (Niel *et al.*, 2003 ▸) and some others have been reported as coligands in these frameworks. Among the above-mentioned azines, the simplest μ_2_-bridging system is pyrazine, which provides 1,4-binding and the formation of compact frameworks (Southon *et al.*, 2009 ▸). Taking into account that the modification of pyrazine can influence not only the structure of a complex but also the spin state of Fe, and being inspired by a previously published structure with 2-bromo­pyrazine as a coligand and bridging cyano­cuprates (Kucheriv *et al.*, 2018 ▸), here we describe the crystal structure of a new Hofmann clathrate analogue of general formula [Fe(Etpz)_2_{Cu(CN)_2_}_2_]_*n*_ (where Etpz is 2-ethyl­pyrazine).
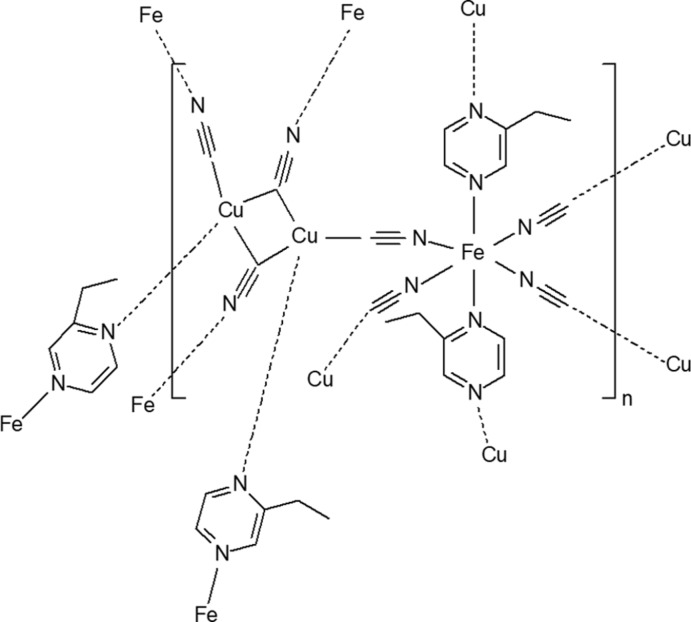


## Structural commentary   

**Figure 1:**
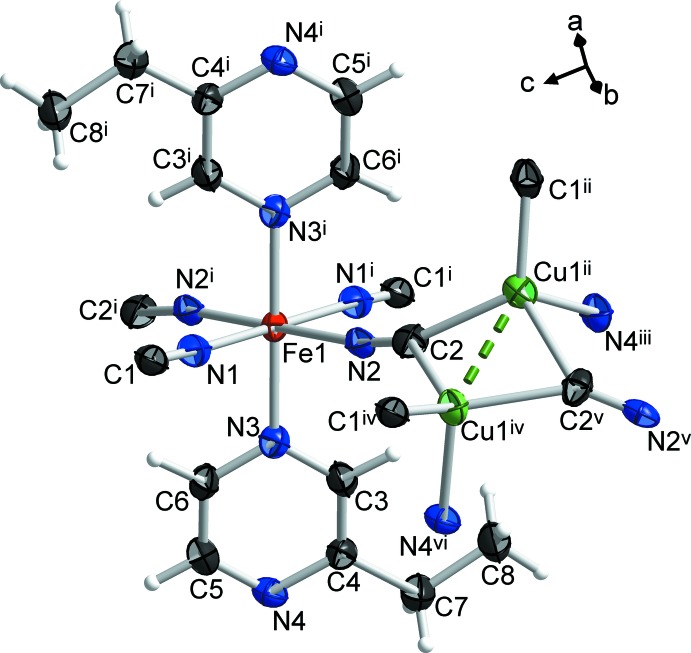
A fragment of the crystal structure of the title compound with atom labelling. Displacement ellipsoids are drawn at the 90% probability level [symmetry codes: (i) 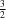 − *x*, 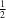 − *y*, 1 − *z*; (ii) *x*, −*y*, −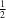 + *z*; (iii) *x*, 1 − *y*, −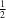 + *z*; (iv) 1 − *x*, −*y*, 1 − *z*; (v) 1 − *x*, *y*, 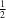 − *z*; (vi) 1 − *x*, 1 − *y*, 1 − *z*]. The Cu⋯Cu short contact is shown as a dashed line.

A fragment of the structure of the title compound is shown in Fig. 1 ▸. The Fe^II^ ion is coordinated *via* N atoms by two pairs of symmetry-related cyanido groups in the equatorial positions [Fe1—N1 = 1.966 (2) and Fe1—N2 = 1.953 (2) Å]. The axial positions are occupied by the N atoms of two symmetry-related 2-ethyl­pyrazine mol­ecules [Fe1—N3 = 1.981 (2) Å]. The low-spin state of the Fe^II^ centre at the temperature of experiment (*T* = 173 K) is confirmed by the Fe—N bond lengths (*i.e.* < 2.0 Å). Each Cu^I^ ion (Cu1^ii^ and Cu1^iv^) is coord­inated by one bridging 2-ethyl­pyrazine mol­ecule *via* the N atom and by the C atoms of three cyanido groups [Cu1^ii^—N4^iii^, Cu1^iv^—N4^vi^ = 2.122 (2), Cu1^ii^—C1^ii^, Cu1^iv^—C1^iv^ = 1.933 (3), Cu1^ii^—C2, Cu1^iv^—C2^v^ = 2.078 (3), Cu1^ii^—C2^v^, Cu1^iv^—C2 = 2.151 (3) Å; symmetry codes: (i) 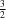 − *x*, 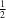 − *y*, 1 − *z*; (ii) *x*, −*y*, −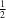 + *z*; (iii) *x*, 1 − *y*, −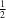 + *z*; (iv) 1 − *x*, −*y*, 1 − *z*; (v) 1 − *x*, *y*, 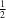 − *z*; (vi) 1 − *x*, 1 − *y*, 1 − *z*]. The separation between two neighboring Cu atoms is 2.4662 (7) Å, which is significantly shorter than the sum of the corresponding van der Waals radii (2.8 Å; Bondi, 1964 ▸), could indicate the presence of metallophilic inter­actions, namely cuprophilic (Hermann *et al.*, 2001 ▸). The Cu atom binds to atom N4 of the 2-ethyl­pyrazine, which is close to the ethyl substituent, while the coordination of the Fe^II^ ion occurs through the more sterically accessible N3 atom of the pyrazine ring.

**Figure 2:**
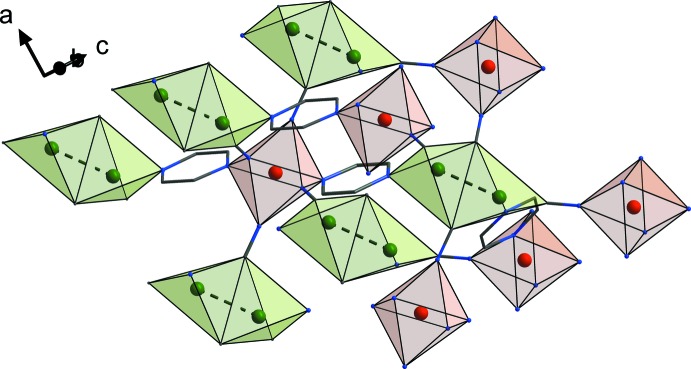
Coordination polyhedra of the Fe and Cu atoms in the title compound. Cu⋯Cu contacts are shown as dashed lines. Colour code: Fe red, Cu green, C grey, N blue.

The coordination polyhedra of Fe and Cu atoms of the title compound and their relative positions are shown in Fig. 2 ▸. Six N atoms form a slightly elongated octa­hedral coordination environment of the Fe^II^ ion. The deviation from an ideal octa­hedron of the Fe1 centre can be described by the octa­hedral distortion parameter Σ|90 − *θ*| = 20.59°, where *θ* is a *cis*-N—Fe—N angle. The fourfold CuC_3_N coordination environment of the Cu^I^ centre adopts a tetra­hedral geometry. Two tetra­hedra of neighboring Cu centres are connected through a common edge between two C atoms of cyanido groups. This common edge is perpendicular to the Cu⋯Cu contact. Each Fe octa­hedron is surrounded by six double Cu–Cu edge-connected tetra­hedra and is bound with them by four cyanido groups and two bridging pyrazine rings. At the same time, dicopper two edge-connected tetra­hedra are linked to four Fe^II^ ion octa­hedrons *via* cyanido bridges and to two Fe octa­hedra *via* pyrazine rings.

## Supra­molecular features   

**Figure 3:**
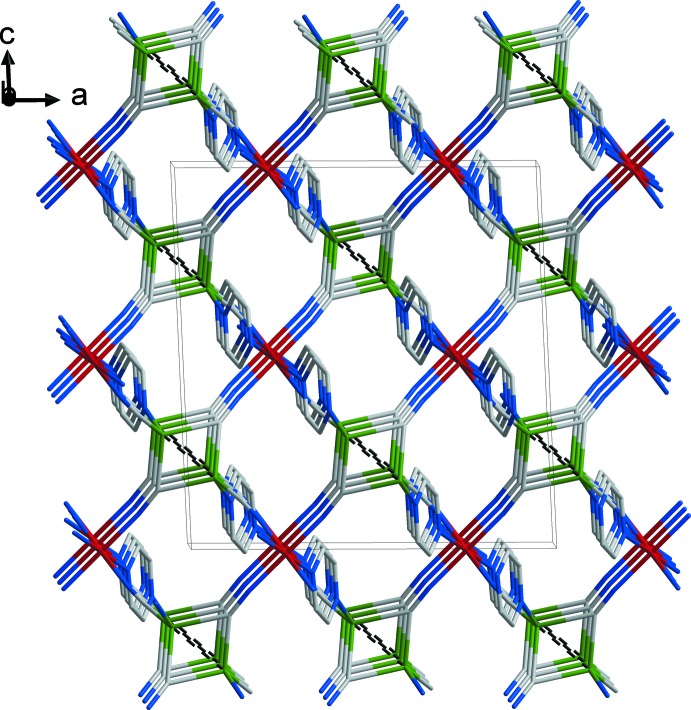
A view normal to the *ac* plane of the crystal structure of the title compound showing the Cu⋯Cu contacts as dashed lines. Ethyl substituents of 2-ethyl­pyrazine rings and H atoms have been omitted for clarity. Colour code: Fe dark red, Cu green, C grey, N blue.

Fig. 3 ▸ illustrates the crystal packing of the title compound. The unit cell contains four units of the title compound with empirical formula C_16_H_16_Cu_2_FeN_8_. The latter consists of bridging 2-ethyl­pyrazine ligands and Cu_2_(CN)_2_ pairs, in which two Cu atoms, centred about a twofold rotation axis, are inter­connected by two μ-CN groups through C atoms. The resulting polymeric three-dimensional metal–organic coordination framework is additionally stabilized by supra­molecular Cu⋯Cu contacts in each Cu_2_(CN)_2_ unit.

## Database survey   

A search through the Cambridge Structural Database (CSD, version 5.40, last update May 2019; Groom *et al.*, 2016 ▸) gave 36 hits for the Cu_2_(CN)_2_ unit, the majority of which are copper monometallic metal–organic frameworks (MOFs). Several bimetallic MOFs are slightly similar to the title compound, namely *catena*-[bis­(μ_3_-chloro)­bis­(μ_3_-cyano)­tetra­kis­(μ_2_-cy­ano)bis­(*N*-methyl­ethane-1,2-di­amine)­dicadmium(II)dicopper(I)copper(II)] (TIDJIB; Kuchár & Černák, 2013 ▸) and *catena*-[bis­(μ_3_-cyano)­tetra­kis­(μ_2_-cyano)­tetra­kis­(di­methyl­formamide­tetra­copper(I)zinc(II)] (UBUROY; Cui *et al.*, 2001 ▸), the structure of which was described as a 3D network with two types of bridging cyanides. The Cu⋯Cu distances are 2.5431 (11) and 2.5734 (13) Å, respectively, compared to 2.4662 (7) Å in the title MOF.

A search through the CSD for the Fe ion ligated by four N≡C–Cu and two azines gave 15 hits, which are all bimetallic MOFs with pyrimidine, cyano­pyridine and fluoro-, chloro-, bromo- and iodo­pyridine as ligands.

A search through the CSD for 2-ethyl­pyrazine gave 20 hits, in most of which 2-ethyl­pyrazine mol­ecule binds to Cu, Ag, Mn or Rh ions. In the majority of compounds containing copper, the 2-ethyl­pyrazine serves as a bridging ligand between two Cu atoms in MOFs. An example closely related to the title structure is *catena*-[(μ_3_-cyano)­tris­(μ_2_-cyano)­bis(μ_2_-2-ethyl­pyrazine)­tetra­copper(I)] (SUYDEV; Chesnut *et al.*, 2001 ▸), in which neighbouring Cu^I^ ions are connected by (i) bridging 2-ethyl­pyrazine mol­ecules and (ii) bridging cyano groups, thus forming one-dimensional {Cu(CN)}_*n*_ chains and double-stranded {Cu(CN)}_*n*_ ribbons, linked into a network by bridging ethyl­pyrazine ligands.

## Synthesis and crystallization   

Crystals of the title compound were obtained by a slow diffusion within three layers in a 3 ml glass tube. The first layer was a solution of K[Cu(CN)_2_] (9.3 mg, 0.06 mmol) in 1 ml of H_2_O; the second layer was an H_2_O/EtOH mixture (1:1, 1 ml); the third layer was a solution of Fe(ClO_4_)_2_·6H_2_O (10.9 mg, 0.03 mmol) and 2-ethyl­pyrazine (6.5 mg, 0.06 mmol) in 0.5 ml of EtOH. After two weeks, brown crystals were formed in the middle layer. The crystals were kept under the mother solution prior to measurement.

## Refinement   

Crystal data, data collection and structure refinement details are summarized in Table 1 ▸. All hydrogen atoms were placed geometrically and refined as riding: C—H = 0.95 Å with *U*_iso_(H) = 1.2*U*_eq_(C) for aromatic hydrogens, C—H = 0.99 Å with *U*_iso_(H) = 1.2*U*_eq_(C) for CH_2_ groups and C—H = 0.98 Å with *U*_iso_(H) = 1.5*U*_eq_(C) for CH_3_ groups.

## Supplementary Material

Crystal structure: contains datablock(s) I. DOI: 10.1107/S2056989019009496/rz5262sup1.cif

Structure factors: contains datablock(s) I. DOI: 10.1107/S2056989019009496/rz5262Isup2.hkl

CCDC reference: 1937912

Additional supporting information:  crystallographic information; 3D view; checkCIF report

## Figures and Tables

**Table 1:** Experimental details

Crystal data	
Chemical formula	[Cu_2_Fe(CN)_4_](C_6_H_8_N_2_)_2_
*M*_r_	503.30
Crystal system, space group	Monoclinic, *C*2/*c*
Temperature (K)	173
*a*, *b*, *c* (Å)	13.1997 (17), 9.2923 (11), 13.8010 (17)
β (°)	92.399 (2)
*V* (Å^3^)	1691.3 (4)
*Z*	4
Radiation type	Mo *K*α
μ (mm^−1^)	3.36
Crystal size (mm)	0.17 × 0.14 × 0.06

Data collection	
Diffractometer	Bruker SMART
Absorption correction	Multi-scan (*SADABS*; Bruker, 2013 ▸)
*T*_min_, *T*_max_	0.614, 0.746
No. of measured, independent and observed [*I* > 2σ(*I*)] reflections	5344, 2022, 1594
*R*_int_	0.065
(sin θ/λ)_max_ (Å^−1^)	0.657

Refinement	
*R*[*F* ^2^ > 2σ(*F* ^2^)], *wR*(*F* ^2^), *S*	0.030, 0.065, 0.93
No. of reflections	2022
No. of parameters	124
H-atom treatment	H-atom parameters constrained
Δρ_max_, Δρ_min_ (e Å^−3^)	0.71, −0.48

Computer programs: *SAINT* and *APEX* (Bruker, 2013 ▸), *SHELXS* (Sheldrick, 2008 ▸), *SHELXL* (Sheldrick, 2015 ▸) and *OLEX2* (Dolomanov *et al.*, 2009 ▸).

## supplementary crystallographic information

### Crystal data

**Table:**

[Cu_2_Fe(CN)_4_](C_6_H_8_N_2_)_2_	*F*(000) = 1008
*M**_r_* = 503.30	*D*_x_ = 1.977 Mg m^−^^3^
Monoclinic, *C*2/*c*	Mo *K*α radiation, λ = 0.71073 Å
*a* = 13.1997 (17) Å	Cell parameters from 1806 reflections
*b* = 9.2923 (11) Å	θ = 2.7–27.8°
*c* = 13.8010 (17) Å	µ = 3.36 mm^−^^1^
β = 92.399 (2)°	*T* = 173 K
*V* = 1691.3 (4) Å^3^	Plate, brown
*Z* = 4	0.17 × 0.14 × 0.06 mm

### Data collection

**Table:**

Bruker SMART diffractometer	1594 reflections with *I* > 2σ(*I*)
ω scan	*R*_int_ = 0.065
Absorption correction: multi-scan (SADABS; Bruker, 2013)	θ_max_ = 27.9°, θ_min_ = 2.7°
*T*_min_ = 0.614, *T*_max_ = 0.746	*h* = −17→17
5344 measured reflections	*k* = −11→12
2022 independent reflections	*l* = −17→18

### Refinement

**Table:**

Refinement on *F*^2^	Primary atom site location: structure-invariant direct methods
Least-squares matrix: full	Hydrogen site location: inferred from neighbouring sites
*R*[*F*^2^ > 2σ(*F*^2^)] = 0.030	H-atom parameters constrained
*wR*(*F*^2^) = 0.065	*w* = 1/[σ^2^(*F*_o_^2^) + (0.0096*P*)^2^] where *P* = (*F*_o_^2^ + 2*F*_c_^2^)/3
*S* = 0.93	(Δ/σ)_max_ = 0.001
2022 reflections	Δρ_max_ = 0.71 e Å^−^^3^
124 parameters	Δρ_min_ = −0.47 e Å^−^^3^
0 restraints	

### Special details

**Table:**

Geometry. All esds (except the esd in the dihedral angle between two l.s. planes) are estimated using the full covariance matrix. The cell esds are taken into account individually in the estimation of esds in distances, angles and torsion angles; correlations between esds in cell parameters are only used when they are defined by crystal symmetry. An approximate (isotropic) treatment of cell esds is used for estimating esds involving l.s. planes.

### Fractional atomic coordinates and isotropic or equivalent isotropic displacement parameters (Å^2^)

**Table:**

	*x*	*y*	*z*	*U*_iso_*/*U*_eq_	
Fe1	0.750000	0.250000	0.500000	0.00838 (13)	
N3	0.67750 (17)	0.4182 (2)	0.55164 (15)	0.0112 (5)	
C6	0.6721 (2)	0.4390 (3)	0.64784 (18)	0.0130 (6)	
H6	0.690965	0.363327	0.691266	0.016*	
Cu1	0.56898 (3)	−0.12263 (4)	0.69244 (2)	0.01200 (10)	
C5	0.6397 (2)	0.5687 (3)	0.68428 (19)	0.0149 (6)	
H5	0.636763	0.579116	0.752586	0.018*	
N4	0.61203 (18)	0.6806 (2)	0.62788 (16)	0.0123 (5)	
C4	0.6133 (2)	0.6591 (3)	0.53050 (18)	0.0116 (6)	
C3	0.6452 (2)	0.5271 (3)	0.49461 (18)	0.0126 (6)	
H3	0.644046	0.513708	0.426328	0.015*	
C7	0.5820 (3)	0.7817 (3)	0.46507 (19)	0.0193 (7)	
H7A	0.621459	0.868000	0.485232	0.023*	
H7B	0.509562	0.802998	0.474233	0.023*	
C8	0.5965 (3)	0.7554 (3)	0.35783 (19)	0.0205 (7)	
H8A	0.574269	0.840394	0.320653	0.031*	
H8B	0.668292	0.736985	0.347349	0.031*	
H8C	0.556167	0.671850	0.336328	0.031*	
N2	0.64347 (17)	0.2077 (2)	0.40162 (15)	0.0104 (5)	
C2	0.5888 (2)	0.1671 (3)	0.33956 (19)	0.0123 (6)	
N1	0.68194 (18)	0.1220 (2)	0.59018 (15)	0.0121 (5)	
C1	0.6394 (2)	0.0361 (3)	0.63471 (18)	0.0130 (6)	

### Atomic displacement parameters (Å^2^)

**Table:**

	*U*^11^	*U*^22^	*U*^33^	*U*^12^	*U*^13^	*U*^23^
Fe1	0.0087 (3)	0.0087 (3)	0.0077 (3)	−0.0002 (2)	−0.00018 (19)	0.0000 (2)
N3	0.0117 (12)	0.0109 (12)	0.0109 (11)	0.0007 (9)	0.0009 (9)	0.0007 (9)
C6	0.0172 (14)	0.0111 (13)	0.0106 (13)	0.0016 (12)	0.0010 (11)	0.0027 (11)
Cu1	0.01269 (17)	0.01261 (18)	0.01078 (17)	−0.00052 (14)	0.00136 (12)	0.00098 (14)
C5	0.0177 (14)	0.0178 (14)	0.0093 (13)	0.0025 (13)	0.0016 (11)	0.0004 (12)
N4	0.0130 (12)	0.0123 (12)	0.0117 (11)	0.0021 (10)	0.0022 (9)	−0.0024 (10)
C4	0.0107 (13)	0.0145 (14)	0.0098 (12)	0.0021 (11)	−0.0001 (10)	0.0002 (11)
C3	0.0138 (13)	0.0154 (15)	0.0087 (13)	−0.0003 (12)	0.0004 (10)	−0.0001 (11)
C7	0.0274 (17)	0.0183 (15)	0.0123 (14)	0.0081 (14)	0.0019 (12)	0.0020 (12)
C8	0.0327 (18)	0.0182 (15)	0.0107 (14)	0.0073 (14)	0.0009 (12)	0.0008 (12)
N2	0.0108 (11)	0.0102 (11)	0.0105 (11)	0.0006 (9)	0.0045 (9)	0.0028 (9)
C2	0.0142 (14)	0.0093 (13)	0.0132 (13)	−0.0001 (11)	−0.0016 (11)	−0.0012 (11)
N1	0.0108 (11)	0.0130 (12)	0.0122 (11)	0.0011 (10)	−0.0020 (9)	−0.0039 (10)
C1	0.0118 (13)	0.0163 (15)	0.0110 (13)	0.0022 (12)	0.0013 (10)	−0.0025 (11)

### Geometric parameters (Å, º)

**Table:**

Fe1—N3	1.981 (2)	C5—H5	0.9500
Fe1—N3^i^	1.981 (2)	C5—N4	1.340 (3)
Fe1—N2	1.953 (2)	N4—C4	1.360 (3)
Fe1—N2^i^	1.953 (2)	C4—C3	1.394 (4)
Fe1—N1^i^	1.966 (2)	C4—C7	1.501 (4)
Fe1—N1	1.966 (2)	C3—H3	0.9500
N3—C6	1.346 (3)	C7—H7A	0.9900
N3—C3	1.341 (3)	C7—H7B	0.9900
C6—H6	0.9500	C7—C8	1.520 (4)
C6—C5	1.381 (4)	C8—H8A	0.9800
Cu1—Cu1^ii^	2.4662 (7)	C8—H8B	0.9800
Cu1—N4^iii^	2.122 (2)	C8—H8C	0.9800
Cu1—C2^iv^	2.151 (3)	N2—C2	1.160 (3)
Cu1—C2^v^	2.078 (3)	N1—C1	1.166 (3)
Cu1—C1	1.933 (3)		
			
N3^i^—Fe1—N3	180.0	C6—C5—H5	118.4
N2—Fe1—N3^i^	86.31 (9)	N4—C5—C6	123.1 (2)
N2^i^—Fe1—N3^i^	93.69 (9)	N4—C5—H5	118.4
N2—Fe1—N3	93.69 (9)	C5—N4—Cu1^vi^	119.72 (18)
N2^i^—Fe1—N3	86.31 (9)	C5—N4—C4	116.5 (2)
N2—Fe1—N2^i^	180.0	C4—N4—Cu1^vi^	123.81 (18)
N2—Fe1—N1^i^	90.94 (9)	N4—C4—C3	119.8 (2)
N2—Fe1—N1	89.06 (9)	N4—C4—C7	118.0 (2)
N2^i^—Fe1—N1	90.94 (9)	C3—C4—C7	122.2 (2)
N2^i^—Fe1—N1^i^	89.06 (9)	N3—C3—C4	123.3 (2)
N1^i^—Fe1—N3	89.48 (9)	N3—C3—H3	118.4
N1^i^—Fe1—N3^i^	90.51 (9)	C4—C3—H3	118.4
N1—Fe1—N3	90.52 (9)	C4—C7—H7A	108.5
N1—Fe1—N3^i^	89.48 (9)	C4—C7—H7B	108.5
N1^i^—Fe1—N1	180.0	C4—C7—C8	114.9 (2)
C6—N3—Fe1	120.93 (19)	H7A—C7—H7B	107.5
C3—N3—Fe1	122.06 (18)	C8—C7—H7A	108.5
C3—N3—C6	116.2 (2)	C8—C7—H7B	108.5
N3—C6—H6	119.5	C7—C8—H8A	109.5
N3—C6—C5	120.9 (3)	C7—C8—H8B	109.5
C5—C6—H6	119.5	C7—C8—H8C	109.5
N4^iii^—Cu1—Cu1^ii^	119.23 (6)	H8A—C8—H8B	109.5
N4^iii^—Cu1—C2^iv^	91.28 (10)	H8A—C8—H8C	109.5
C2^v^—Cu1—Cu1^ii^	55.71 (8)	H8B—C8—H8C	109.5
C2^iv^—Cu1—Cu1^ii^	52.95 (7)	C2—N2—Fe1	170.6 (2)
C2^v^—Cu1—N4^iii^	102.36 (10)	Cu1^vii^—C2—Cu1^iv^	71.33 (8)
C2^v^—Cu1—C2^iv^	104.11 (10)	N2—C2—Cu1^vii^	146.7 (2)
C1—Cu1—Cu1^ii^	130.26 (8)	N2—C2—Cu1^iv^	141.4 (2)
C1—Cu1—N4^iii^	110.04 (10)	C1—N1—Fe1	172.3 (2)
C1—Cu1—C2^iv^	122.70 (11)	N1—C1—Cu1	172.0 (2)
C1—Cu1—C2^v^	120.75 (11)		
			
Fe1—N3—C6—C5	167.2 (2)	C5—N4—C4—C3	−1.9 (4)
Fe1—N3—C3—C4	−166.3 (2)	C5—N4—C4—C7	179.5 (3)
N3—C6—C5—N4	−0.1 (5)	N4—C4—C3—N3	−1.4 (4)
C6—N3—C3—C4	3.8 (4)	N4—C4—C7—C8	173.5 (3)
C6—C5—N4—Cu1^vi^	−177.6 (2)	C3—N3—C6—C5	−3.1 (4)
C6—C5—N4—C4	2.7 (4)	C3—C4—C7—C8	−5.0 (4)
Cu1^vi^—N4—C4—C3	178.3 (2)	C7—C4—C3—N3	177.1 (3)
Cu1^vi^—N4—C4—C7	−0.2 (4)		

Symmetry codes: (i) −*x*+3/2, −*y*+1/2, −*z*+1; (ii) −*x*+1, *y*, −*z*+3/2; (iii) *x*, *y*−1, *z*; (iv) −*x*+1, −*y*, −*z*+1; (v) *x*, −*y*, *z*+1/2; (vi) *x*, *y*+1, *z*; (vii) *x*, −*y*, *z*−1/2.
